# The impact of interpersonal racism on oral health related quality of life among Indigenous South Australians: a cross-sectional study

**DOI:** 10.1186/s12903-021-01399-1

**Published:** 2021-02-04

**Authors:** Anna Ali, Alice R. Rumbold, Kostas Kapellas, Zohra S. Lassi, Joanne Hedges, Lisa Jamieson

**Affiliations:** 1 Robinson Research Institute, The University of Adelaide, 30 Frome Road, Adelaide, SA 5000 Australia; 2 South Australian Health and Medical Research Institute, North Terrace, Adelaide, SA 5000 Australia; 3 Australian Research Centre for Population Oral Health, Adelaide Health and Medical Sciences, The University of Adelaide, 4 North Terrace, Adelaide, 5005 Australia

**Keywords:** Oral health related quality of life, Racism, Indigenous Australians, Aboriginals, Torres Strait Islanders

## Abstract

**Background:**

Interpersonal racism has had a profound impact on Indigenous populations globally, manifesting as negative experiences and discrimination at an individual, institutional and systemic level. Interpersonal racism has been shown to negatively influence a range of health outcomes but has received limited attention in the context of oral health. The aim of this paper was to examine the effects of experiences of interpersonal racism on oral health-related quality of life (OHRQoL) among Indigenous South Australians.

**Methods:**

Data were sourced from a large convenience sample of Indigenous South Australian adults between February 2018 and January 2019. Questionnaires were used to collect data on sociodemographic characteristics, cultural values, utilization of dental services, and other related factors. OHRQoL was captured using the Oral Health Impact Profile (OHIP-14) questionnaire. We defined the dependent variable 'poor OHRQoL' as the presence of one or more OHIP-14 items rated as ‘very often’ or ‘fairly often'. Experiences of racism were recorded using the Measure of Indigenous Racism Experiences instrument. Interpersonal racism was classified into two categories (‘no racism’ vs ‘any racism in ≥ 1 setting’) and three categories ('no racism', 'low racism' (experienced in 1–3 settings), and 'high racism' (experienced in 4–9 settings)). Logistic regression was used to examine associations between interpersonal racism, covariates and OHRQoL, adjusting for potential confounding related to socioeconomic factors and access to dental services.

**Results:**

Data were available from 885 participants (88.7% of the total cohort). Overall, 52.1% reported experiencing any interpersonal racism in the previous 12 months, approximately one-third (31.6%) were classified as experiencing low racism, and one-fifth (20.5%) experienced high racism. Poor OHRQoL was reported by half the participants (50.2%). Relative to no experiences of racism in the previous 12 months, those who experienced any racism (≥ 1 setting) were significantly more likely to report poor OHRQoL (Odds Ratio (OR): 1.43; 95% Confidence Interval (CI): 1.08–1.92), after adjusting for age, education level, possession of an income-tested health care card, car ownership, self-reported oral health status, timing of and reason for last dental visit, not going to a dentist because of cost, and having no family support. This was particularly seen among females, where, relative to males, the odds of having poor OHRQoL among females experiencing racism were 1.74 times higher (95% CI: 1.07–2.81).

**Conclusion:**

Our findings indicate that the experience of interpersonal racism has a negative impact on OHRQoL among Indigenous Australians. The association persisted after adjusting for potential confounding factors. Identifying this link adds weight to the importance of addressing OHRQoL among South Australian’s Indigenous population by implementing culturally-sensitive strategies to address interpersonal racism.

## Background

Racism has been defined as anything “that which maintains or exacerbates inequality of opportunity among ethno-racial groups” [1]. Racism can have a profound impact on society through exclusion and marginalisation of certain ethnic or minority groups and promotion of concepts of inferiority in the worldview of the society as a whole [2–4]. At an individual level, numerous studies demonstrate that racism can have a deleterious effect on health. This has been most studied in the context of adverse mental health outcomes and health behaviours [5–7]. The effects of racism on health may occur via several pathways including chronic stress and distrust of health care providers, which may reduce health care seeking behaviours and use of preventive services [8].

In a healthcare setting, racial discrimination is considered to be an individual’s appraisal of unfair treatment related to differences in race, appearance and ethnicity [9]. Theories explaining racism as a contributor to health inequalities argue that racism can act on three levels (institutionalized, personal, and internalized) to influence health outcomes [10]. For example, institutional racism arising from unfair distribution of goods, services, and opportunities could lead to unfair and differential access to health-promoting resources [11]. It could also influence health care providers’ decision making, treatment strategies and communication, through the development of implicit racial bias and explicit racial stereotypes [12, 13]. Another pathway through which racism can impact on health outcomes is via psychological stress. Racism may act as a stressor that, in turn, results in psychological harm (e.g. distress), and physiological changes (e.g. increased blood pressure, pulse rate, nervousness, nausea), whilst simultaneously causing changes in health-seeking behaviours [14–16]. All factors may cumulatively provoke involuntary responses, such as anxiety or increased vigilance and voluntary coping responses including disengagement from situations or environments that negatively stereotype individuals, including health care settings [17].

The impacts of racism are disproportionately felt by the most disadvantaged social groups. This includes many Indigenous peoples, who have been profoundly affected by the ongoing effects of systemic and interpersonal racism [6, 18, 19]. Indigenous Australians, those who identify as being of Aboriginal and/or Torres Strait Islander descent [20], are the oldest continuing civilization, dating back more than 65,000 years [21]. Following European colonisation of Australia in the late 1700s, population numbers of Indigenous Australians rapidly declined due to extensive dispossession of land, massacres and relocation of people into religious missions [22]. The effects of these practices are still felt today, and manifest in much higher rates of social, economic and health disadvantage in many Indigenous communities. This includes experiences of marginalisation and racism, which have been reported at interpersonal, institutional and broader structural levels [23]. Indeed there is increasing recognition that both historic and contemporary racism contributes to longstanding inequalities in health, income, housing, and education between Indigenous and non-Indigenous Australians [24]. Notably, while racism is experienced by Indigenous men and women, the effects of racism are gendered, exemplified by experiences of family violence and homelessness disproportionately affecting Indigenous women [25].

Poor oral health is a major concern for Indigenous Australians, who experience very high rates of untreated dental disease including dental caries, gum disease, tooth loss and tooth ache relative to non-Indigenous Australians [26]. Further, a study conducted during 2014–2015 among Indigenous Australians found females were more likely than  males to report oral conditions such as dental caries, utilisation of dentures, and pain/discomfort in the teeth or mouth [27]. Poor oral health can have a significant impact on overall health as well as quality of life, affecting the ability to eat, speak and sleep, as well as confidence with social and professional interactions. As a result, poor oral health can influence productivity in the workplace and negatively influence social and emotional well-being [28].

Oral health-related quality of life (OHRQoL) is an important measure of the impact of oral conditions on health-related well‐being [28]. OHRQoL encompasses a range of factors such as access and utilisation of health services, caregiver characteristics, and sociodemographic characteristics that could influence oral health [28]. The broad and complex multidimensional construct of OHRQoL enables the examination of the impact of oral health on psychological, functional, and social functioning [28]. As experiences of racism can also affect the psychological and physiological well-being of an individual, it is plausible that racism could influence OHRQoL. However, this has received limited research attention. We identified only one published study conducted in Canada among pregnant Aboriginal women, which reported an adverse impact of racism on OHRQoL [5]. We have previously shown that racism experienced by Indigenous Australians is associated with poorer oral health behaviours such as tooth brushing and use of dental services [29–31]. Both are likely to affect OHRQoL, however, to date no study has examined the possible effects of racism on OHRQoL among Indigenous Australians.

The main aim of this study was to explore the association between self-reported interpersonal racism and OHRQoL among Indigenous South Australians, adjusting for potential confounding by socioeconomic factors. Our goal was to examine these relationships in a population with high levels of untreated dental disease, to understand the potential widespread impacts of racism and  inform health and social policy to address racial and oral health inequalities.

## Methods

### Study design and participants

This paper is a part of a larger study investigating oral human papillomavirus infection and oropharyngeal squamous cell carcinomas (OPSCC) among Indigenous Australians [32]. The study is governed by an Indigenous Reference Group, with data collected by trained Indigenous research officers. Data for this paper are drawn from the baseline data collection (Additional file 1: Table S1), which includes a large convenience sample of Indigenous South Australians recruited between February 2018 and January 2019.

### Self-reported variables

Sociodemographic characteristics collected included age, gender, geographic location, highest completed education level, current employment status, ownership of a means-tested government health care card, number of people living in household on the previous night, and car ownership. Health-related behaviours assessed included tobacco and alcohol use. Information indicating oral health status and use of dental services was also collected. This included self-rated oral health (excellent, good, fair and poor), timing of last dental visit (less than a year ago or more than a year ago), dental cost factors (not attending a dentist due to cost, difficulty in paying a $100 AUD dental bill), and reason for last dental visit (problem vs. check-up).

Information was also collected on aspects of cultural identity, by asking the following questions which were assessed on a Likert scale: (1) Do you know a lot about your Aboriginal/Torres Strait Islander culture? (2) Do you identify with a tribal group, a language group or clan? (3) To you, is being Aboriginal/Torres Strait Islander the most important thing or important but not the only thing or want to know more or something you don’t think about? (4) Do you feel like you know a lot about white fella ways? (5) Do you have a strong family who help each other?

### Outcome: oral health-related quality of life (OHRQoL)

To evaluate OHRQoL, the short form of the oral health impact profile—OHIP-14 questionnaire was used [33]. This contains 14 items relating to the frequency with which oral conditions adversely affect quality of life. OHIP is assessed on a five-point ordinal scale with responses: very often (4), often (3), occasionally (2), hardly ever (1), and never (0). The first four items ask participants how often in the last year had they experienced 'toothache or pain in the mouth', 'bleeding gums when brushing', 'chronic dry mouth', and 'chronic bad breath'. The following three items evaluated the frequency with which problems with teeth, dentures or gums produced varying levels of psychological discomfort (uncomfortable at work, school or in social situations), functional limitation (not able to eat some foods or had to eat them slowly), and social disability (missed work, school or took time away from normal activities) (Additional file 1: Tables S1 and S2). This tool has been validated among Indigenous and non-Indigenous Australians [34]. We defined the dependent variable 'poor OHRQoL' as the presence of one or more OHIP-14 items rated ‘very often’ or ‘fairly often' [35].

### Primary exposure: experience of interpersonal racism

Interpersonal racism was assessed using an adapted version of the Measure of Indigenous Racism Experiences (MIRE) instrument across nine settings [36]. This version was shorter than the original [36], and was concerned with whether participants experienced unfair treatment due to their race in those nine settings over the last 12 months. Participants were asked: 'In the last twelve months, have you felt that you have been treated unfairly in any of the following ways because you are Aboriginal?' The settings included: employment, domestic, educational/academic, recreational/leisure, law (enforcement), health care, government service provision, other service provision, public, any other situation (re-categorised according to the other settings where relevant). Response options were ‘yes’ or ‘no’. Experience of interpersonal racism was computed as a summary score; range 0–9 [29]. The summary score was dichotomised into two ways. First, the experience of 'any racism' (reported in ≥ 1 setting) vs no racism; and second, experience of 'low racism' (reported in 1–3 settings), 'high racism' (reported in 4–9 settings), and 'no racism'.

### Statistical analysis

Normality of all continuous variables was assessed. Variables that were not normally distributed were re-coded as a categorical variable. Collinearity was assessed with no variables needing to be excluded due to weak associations. The frequency and percentage of categorical variables, and mean and standard deviation of continuous variables were reported. Variables conceptually associated with OHRQoL and racism were explored as potential confounders in the analyses after evaluating recent empirical evidence. Previous literature has shown strong associations between sociodemographic  characteristics, health-related behaviours and use of dental services with OHRQoL and interpersonal racism among Indigenous Australians [28, 29, 35]. For example, a study conducted among Indigenous Australians in 2009 reported significant associations between factors such as age, use of a health care card, smoking and alcohol with poor OHRQoL. With respect to dental service use and oral health status, factors such as problem-based dental attendance (rather than attendance for preventive health), avoiding dental care because of cost of care, and reported difficulty paying a AUD $100 dental bill were also significantly associated with poor OHRQoL [35].

Bivariate associations between sociodemographic characteristics, cultural identity variables, dental service-related  and oral health-related covariates and (1) OHRQoL (OHIP-14), and separately, (2) experience of interpersonal racism classified as ‘no racism’ vs ‘any racism in ≥ 1 setting(s)’ and then ‘no racism’ vs ‘low racism’ and ‘high racism', were explored and tested for statistical significance using logistic regression. All variables with a *p value* ≤ 0.25 were considered as a potential confounder or interaction variable. The association between experiences of racism and OHRQoL is reported as an odds ratio (OR) with 95% confidence intervals (CI). Multivariable logistic regression models were used to produce covariate-adjusted OR and 95% CI. To select the final variables for inclusion in the models, we included all candidate variables (sociodemographic characteristics, cultural identity variables, dental services use and oral health-related) in the model and then applied purposeful backward elimination as described by Hosmer and Lemeshow [37, 38], until the model contained only variables significant at *p* ≤ 0.05. Potential interactions were explored between the variables racism ('no racism' vs 'any racism') and gender and age (≤40 vs >40).

Four different models were generated. Model 1 represents the association between experiences of interpersonal racism classified as 'low racism', 'high racism' vs 'no racism') and OHRQoL. Model 2 represents the association between experiences of interpersonal racism classified as ‘no racism’ vs 'any racism' with OHRQoL. Both Model 1 and Model 2 include adjustment for education level, age, possession of a health care card, car ownership, self-reported oral health status, timing of last dental visit, reason for last dental visit, not attending a dentist due to cost, and level of family support, with significance set at *p* ≤ 0.05. Models 3 and 4 examine the association between ‘no racism’ vs ‘any racism’ with OHRQoL adjusting for the same variables included in Model 2 plus gender (Model 3), and separately  an interaction term between the variables racism (‘no racism vs ‘any racism’) and gender (Model 4). Model performance was evaluated by estimating model discrimination (c-statistic) and calibration. All analyses were conducted using STATA 15.

## Results

Of the total 1011 participants, data from 885 (87.5%) participants were available on experiences of interpersonal racism. Just under one-third (31.6%) experienced low racism (e.g. reported in 1–3 settings in the past 12 months), while one-fifth (20.5%) experienced high racism (e.g. reported in 4–9 settings). The most frequent setting where participants reported racism was law enforcement (31.7%), followed by public settings (24.8%), and government services (22.0%) (Table 1).

**Table 1:** Frequency of each Measure of Indigenous Racism Experiences (MIRE) item and summary racism score (N = 885)

Individual settings where racism was experienced ^a^	n (%)	95% CI
Employment	161(19.2)	15.70–20.89
Domestic	118(13.8)	11.16–15.75
Educational/academic	143(17.0)	13.79–18.75
Recreational/leisure	139(16.2)	13.37–18.27
Law (enforcement)	271(31.7)	27.60–33.78
Health care	151(17.4)	14.64–19.71
Government service provision	191(22.0)	18.91–24.44
Other service provision	154(17.8)	14.96–20.06
Public settings	216(24.8)	21.61–27.38
*Interpersonal racism summary score*		
No racism	424(47.9)	44.57–51.26
Low racism (1–3 settings)	280(31.6)	28.58–34.82
High racism (4–9 settings)	181(20.5)	17.84–23.36
Any racism (≥ 1 setting)	461(52.1)	48.74–55.43

^a^Participants were asked: “In the last twelve months, have you felt that you have been treated unfairly in any of the following ways because you are Aboriginal?” The settings were: (1) Applying for work or when at work; (2) At home, by neighbors or at somebody else’s house; (3) At school, university, training course, or other educational setting; (4) While doing any sporting, recreational or leisure activities; (5) By the police, security people, lawyers or in a court of law; (6) By doctors, dentists, nurses or other staff at hospitals, dental clinics or doctor’s surgeries; (7) By staff of government agencies; (8) When seeking any other services; (9) By members of the general public; (10) Any other situation (re-categorized according to the other 9 settings where relevant)

Regarding the sociodemographic characteristics of study participants, more than half were aged 40 years or younger (55.8%), approximately two-thirds were female (67.0%) and had attained a high school-level education (68.0%), whilst 74.9% were on welfare and held a health care card (78.3%). The majority of study participants had never smoked (57.7%) (Table 2). More than one-third  reported 'a lot' (36.5%) and 'a fair bit' (39.4%) of knowledge of being Aboriginal (Table 3). More than two-thirds (69.5%) identified with a tribal group. For more than half of the participants, being Aboriginal was reported as 'the most important thing' (55.6%) and for 31.2% it was 'important but not the only thing'. More than one third reported that they knew 'a lot' (42.8%) or  had a 'fair bit' (40.1%) of knowledge about white fellas (Western). Around two-thirds (61.6%) of participants reported that they always had  strong family support.

**Table 2:** Bivariate associations between sociodemographic and health-related variables with OHRQoL and interpersonal racism (N = 885)

Sociodemographic and health-related variables	n (%)	OHRQoL, n (%)		Interpersonal racism, n (%)			Poor OHRQoL
		Good (n = 440)	Poor (n = 445)	No racism (n = 424)	Low racism (n = 280)	High racism (n = 181)	Unadjusted OR (95%CI)
*Age in years*							
≤40	494(55.8)	265(53.6)	229(46.4)	237(48.0)	174(35.2)	83(16.8)	Reference (Ref)
> 40	391(44.2)	175(44.8)	216(55.2)^b^	187(47.8)	106(27.1)	98(25.1)	1.43(1.09–1.86)^c^
*Gender*							
Female	593(67.0)	286(48.2)	307(51.8)	310(52.3)	169(28.5)	114(19.2)	Ref
Male	292(33.0)	154(52.7)	138(47.3)	114(39.0)	111(38.0)	67(22.9)	0.84(0.63–1.11)^c^
*Completed education level*							
University or further	283(32.0)	153(54.1)	130(45.9)	117(41.3)	99(35.0)	67(23.7)	Ref
High school or less	602(68.0)	287(47.7)	315(52.3)	307(51.0)	181(30.1)	114(18.9)	1.29(0.97–1.71)^c^
*Employment status*^a^							
Employed	221(25.1)	126(57.0)	95(43.0)	101(45.7)	71(32.1)	49(22.2)	Ref
On welfare/Other	659(74.9)	313(47.5)	346(52.5)^b^	322(48.9)	207(31.4)	130(19.7)	1.47(1.08–1.99)^c^
*Health care card possession*							
No	192(21.7)	110(57.3)	82(42.7)	89(46.4)	64(33.3)	39(20.2)	Ref
Yes	693(78.3)	330(47.6)	363(52.4)^b^	335(48.3)	216(31.2)	142(20.6)	1.45(1.07–2.04)^c^
*No. of people who stayed in house last night*							
≤4	518(58.5)	260(50.2)	258(49.8)	253(48.8)	152(29.3)	113(21.8)	Ref
> 4	367(41.5)	180(49.0)	187(51.0)	171(46.6)	128(34.9)	68(18.5)	1.05(0.80–1.37)
*Car ownership*							
Yes	490(55.4)	261(45.3)	229(46.7)	226(46.1)	160(32.7)	104(21.2)	Ref
No	395(44.6)	179(45.3)	216(54.7)^b^	198(50.1)	120(30.4)	77(19.5)	1.38(1.05–1.80)^c^
*Tobacco smoking*^a^							
Never/don’t know	253(29.0)	146(57.7)	107(42.3)	146(57.7)	64(25.3)	43(17.0)	Ref
Current/previous smoker	620(71.0)	287(46.3)	216(54.7)^b^	273(44.0)	212(34.2)	135(21.8)	1.58(1.18–2.13)^c^
*Alcohol intake*^a^							
Never	317(36.5)	151(47.6)	166(52.4)	165(52.1)	90(28.4)	62(19.6)	Ref
Current/previous consumption	551(63.5)	278(50.5)	273(49.5)	249(45.2)	186(33.8)	116(21.1)	0.89(0.68–1.18)

^a^Indicates some missing data

^b^Poor OHRQoL versus good OHRQoL: *p* value for chi^2^:  ≤ 0.05

^c^Poor OHRQoL versus good OHRQoL: *p* value for univariate logistic regression:  ≤ 0.25

**Table 3:** Bivariate associations between cultural identity variables with OHRQoL and interpersonal racism (N = 885)

Cultural identity and related variables	n (%)	OHRQoL, n (%)		Interpersonal racism, n (%)			Poor OHRQoL
		Good (n = 440)	Poor (n = 445)	No racism (n = 424)	Low racism (n = 280)	High racism (n = 181)	Unadjusted OR (95% CI)
*Know a lot about your Aboriginal/Torres Strait Islander culture*^a^							
Little bit/not much	212(24.1)	104(49.1)	108(50.9)	121(57.1)	60(28.3)	31(14.6)	Ref
A lot/fair bit	669(75.9)	335(50.1)	334(49.9)	302(45.1)	219(32.7)	148(22.1)	1.04(0.76–1.42)^c^
*Do you identify with a tribal group, a language group or clan*?^a^							
No	148(16.9)	69(46.6)	79(53.4)	87(58.8)	39(26.4)	22(14.9)	Ref
Don’t know	114(13.0)	55(48.2)	59(51.8)	62(54.4)	30(26.3)	22(19.3)	
Yes	615(70.1)	313(50.9)	302(49.1)	272(44.2)	207(33.7)	136(22.1)	0.86(0.64–1.15)
*To you, is being Aboriginal/Torres Strait Islander*^a^							
Something you don’t know enough about and want to know more about/Something you don’t think about	115(13.1)	51(44.3)	64(55.7)	66(57.4)	30(26.1)	19(16.5)	Ref
The most important thing/Important, but not the only thing	766(86.9)	386(50.4)	380(49.6)	356(46.5)	249(32.5)	161(21.0)	0.78(0.53–1.16)^c^
*Know a lot about white fella ways*^a^							
Little bit/not much	150(17.0)	74(49.3)	76(50.7)	89(59.3)	40(26.7)	21(14.0)	Ref
A lot/fair bit	733(83.0)	364(49.7)	369(50.3)	334(45.6)	239(32.6)	160(21.8)	0.99(0.69–1.40)
*Have a strong family who help each other*							
Always/most times	748(84.5)	387(51.7)	361(48.3)	370(49.5)	229(30.6)	149(19.9)	Ref
Sometimes/not really	137(15.5)	53(38.7)	84(61.3)^b^	54(39.4)	51(37.2)	32(23.4)	1.70(1.18–2.57)^c^

^a^Indicates some  missing data

^b^Poor OHRQoL versus good OHRQoL: *p* value for chi^2^: ≤ 0.05

^c^Poor OHRQoL versus good OHRQoL: *p* value for univariate logistic regression:  ≤ 0.25

Regarding oral health, half (50.3%) the participants reported poor OHRQoL (OHIP-14 scores very often and often) (Table 2). Just over half of all participants reported their last dental visit was more than a year ago (53.4%) and that the visit for a dental problem (61.8%). Around one-third reported that they avoided dental care because of the  cost (32.3%) and 44.7% reported they would have difficulty paying a $100 AUD dental bill. Two-thirds rated their oral health as excellent/good (Table 4).

In bivariate analysis, poor OHRQoL was associated with age > 40 years, being on welfare, possession of a health care card, ownership of car, and being a current or past smoker (Table 2). Poor OHRQoL was additionally associated with not having strong family support (OR 1.70; 95% CI: 1.18–2.57) (Table 3). In terms of self-rated oral health and dental service utilisation, poor OHRQoL was significantly associated with a dental consultation for a dental problem (OR 2.50; 95% CI: 1.90–3.31), avoiding a dental visit because of the cost (OR 1.88; 95% CI 1.41–2.50), difficulty in paying a $100 AUD dental bill (OR 1.69; 95% CI: 1.21–2.17), and fair or poor self-rated oral health (OR 3.43; 95% CI: 2.55–4.62) (Table 4).

**Table 4:** Bivariate associations between dental service utilisation and OHRQoL and interpersonal racism (N = 885)

Dental services and oral health related quality of life	n (%)	OHRQoL, n (%)		Interpersonal racism, n (%)			Poor OHRQoL
		Good (n = 440)	Poor (n = 445)	No racism (n = 424)	Low racism (n = 280)	High racism (n = 181)	Unadjusted OR (95% CI)
*Timing of last dental visit *							
Less than a year ago	412(46.6)	196(47.6)	216(52.4)	196(47.6)	141(34.2)	75(18.2)	Ref
More than a year ago	473(53.4)	244(51.6)	229(48.4)	228(48.2)	139(29.4)	106(22.4)	0.85(0.65–1.11)^c^
*Reason for last dental visit *^a^							
Check up	338(38.2)	215(63.6)	123(36.4)	190(56.2)	95(28.1)	53(15.7)	Ref
Problem	547(61.8)	225(41.1)	322(58.9)^b^	234(42.8)	185(33.8)	128(23.4)	2.50(1.90–3.31)^c^
*Avoidance of dental care because of cost*							
No	599(67.7)	328(54.8)	271(45.2)	297(49.6)	183(30.6)	119(19.9)	Ref
Yes	286(32.3)	112(39.2)	174(60.8)^b^	127(44.4)	97(33.9)	62(21.7)	1.88(1.41–2.50)^c^
*Level of difficulty paying an AUD$100 dental bill*^a^							
None, hardly any, a little	484(55.3)	265(54.8)	219(45.2)	251(51.9)	155(32.0)	78(16.1)	Ref
A lot	392(44.7)	170(43.4)	222(56.6)^b^	168(42.9)	124(31.6)	100(25.5)	1.69(1.21–2.17)^c^
*Self-rated oral health*							
Excellent, very good, or good	589(66.6)	351(59.6)	238(40.4)	305(51.8)	183(31.1)	101(17.1)	Ref
Fair or poor	296(33.4)	89(30.1)	207(69.9)^b^	119(40.2)	97(32.8)	80(27.0)	3.43(2.55–4.62)^c^

^a^Indicates some missing data

^b^Poor OHRQoL versus good OHRQoL: *p* value for chi^2^:  ≤ 0.05

^c^Poor OHRQoL versus good OHRQoL: *p* value for univariate logistic regression:  ≤ 0.25

There was a positive association between poor OHRQoL and experiences of low racism (OR 1.59; 95% CI: 1.17–2.15) and high racism (OR 1.73; 95% CI: 1.22–1.46) relative to those who reported no racism (Table 5). The association between poor OHRQoL and low racism persisted after adjusting for education level, age, possession of a health care card, car ownership, self-reported oral health status, timing and reason for last dental visit, cost associated with dental visit and family support (OR 1.45; 95% CI: 1.04–2.03) (Table 6).

**Table 5:** Crude associations between interpersonal racism and poor OHRQoL (N = 885)

Interpersonal racism	n (%)	OHRQoL, n (%)		Poor OHRQoL
		Good (n = 440)	Poor (n = 445)	Unadjusted OR (95%CI)
*Interpersonal racism (no, low or high)*				
No racism	424(47.9)	238(56.1)	186(43.9)	Ref
Low racism	280(31.6)	125(44.6)	155(55.4)	1.59(1.17–2.15)
High racism	181(20.2)	77(42.5)	104(57.5)^a^	1.73(1.22-.46)
*Interpersonal racism (no or any)*				
No racism	424(47.9)	238(56.1)	186(43.9)	Ref
Any racism	461(52.1)	202(43.8)	259(56.2)^a^	1.64(1.25–2.14)
Interpersonal racism*gender				
No racism: male	114(26.8)	67(58.8)	47(41.2)	Ref
No racism: female	310(73.1)	171(55.2)	139(44.8)	1.15(0.75–1.79)
Any racism: male	178(38.6)	87(48.9)	91(51.1)	1.49(0.92–2.39)
Any racism: female	283(61.3)	115(40.6)	168(59.4)^a^	2.08(1.33–3.23)
Interpersonal racism*age				
No racism: ≤ 40	237(55.9)	140(59.1)	97(40.9)	Ref
No racism: > 40	187(44.1)	98(52.4)	89(47.6)	1.31(0.89–1.92)
Any racism: ≤ 40	257(55.7)	125(48.6)	132(51.4)^a^	1.52(1.06–2.17)
Any racism: > 40	204(44.2)	77(37.7)	127(62.3)^a^	2.38(1.62–3.49)

^a^Poor OHRQoL versus good OHRQoL: *p* value for chi^2^ ≤ 0.05

**Table 6:** Multivariate models examining the association between interpersonal racism and poor OHRQoL with adjustment for potential confounding factors (N = 885)

	Model 1	Model 2	Model 3	Model 4
LR chi^2^	143.54	143.51	144.98	145.10
Pseudo R^2^	0.1170	0.1170	0.1182	0.1183
Log likelihood	-541.650	-541.066	-540.93	-540.87
Area under curve (AUC)	72.6%	72.6%	72.7%	72.7%
Hosmer and Lemeshow chi^2^	8.78 (*p* value = 0.360)	9.29 (*p* value = 0.318)	8.74 (*p* value = 0.364)	8.35 (*p* value = 0.40)
Low racism	1.45 (95% CI:1.04–2.03)			
High racism	1.40 (95% CI: 0.95–2.06)			
No racism	Ref			
AOR of any racism and poor OHRQoL		1.43(95% CI:1.08–1.92)	1.48(95% CI:1.09–1.98)	
No racism: female				1.14(95% CI: 0.71–1.82)
Any racism: male				1.37(95% CI: 0.82–2.30)
Any racism: female				1.74(95% CI: 1.07–2.81)
No racism: male				Ref

Model 1: Adjusted for education level, age, possession of a health care card, car ownership, self-reported oral health status, timing of and reason for last dental visit, not going to the dentist because of cost and no family support

Model 2: Adjusted for education level, age, possession of a health care card, car ownership, self-reported oral health status, timing of and reason for last dental visit, not going to the dentist because of cost and no family support

Model 3: Adjusted for variables in Model 2 and gender

Model 4: Adjusted for variables in Model 2 and the interaction term (gender*racism)

An association between poor OHRQoL and racism was also present when racism was categorised as 'any racism' (≥ 1 settings) compared with 'no racism' (OR 1.64; 95% CI: 1.25–2.14) (Table 5). The experience of any racism persisted as a risk factor for poor OHRQoL after adjusting for education level, age, possession of a health care card, car ownership, self-reported oral health status, timing of and reason for last dental visit, not going to the dentist because of cost and level of family support (Table 6). The association was strengthened when gender was added as a covariate (AOR 1.48; 95% CI: 1.09–1.98). An interaction term (any racism*gender) was added in multivariate models and a significant interaction between being female with racism was found. The odds of poor OHRQoL were 1.74 times (95% CI: 1.07–2.81) higher among females experiencing any racism compared to males experiencing no racism (Table 6). All four risk adjustment models had moderate discrimination (C-statistic of 0.72) and closely approximated the observed risk suggesting good model calibration (Additional file 1: Fig. S3).

## Discussion

In this study we found that individuals who reported experiencing interpersonal racism were more likely to have poor OHRQoL. The association was strongest among those who experienced racism in four or more settings (classified as 'high racism'), and among women. The associations held after adjusting for sociodemographic and cultural identity characteristics including education and measures of socioeconomic status, as well as factors related to use of dental services. 

Previous research conducted among Indigenous populations has found strong associations between interpersonal racism and different aspects of oral health such as increased early childhood caries, less frequent tooth brushing and less frequent visits to dental services [5, 29, 39]. However, the association between interpersonal racism and OHRQoL has received limited attention previously. Interpersonal racism may impact OHRQoL via several pathways. Experiences of racism may cause cognitive, emotional and physical signs of stress, contributing to poor mental and psychosocial well-being [40]. Further, these experiences could negatively impact on adaptive behaviours (regularly tooth brushing even when feeling anxious or stressed) and promote certain maladaptive behaviours (increased tobacco smoking and alcohol consumption) [40]. The feeling of inferiority induced by experiencing interpersonal racism, especially across multiple settings, could lead to dental fear and anxiety, which may in turn lead to avoidance of services and deterioration in dental status [41]. It has been argued that those who enter this vicious cycle, visit the dentist only when necessary (because of an existing problem) and not for general preventive care. This is also evident in our findings, where more than half of the individuals who faced any kind of interpersonal racism reported going to consult the dentist because of an existing problem and not for a routine check-up.

Experiences of interpersonal racism could also impact on an individual’s sense of coherence (SOC). This has been proposed to underpin positive health-related behaviours as high levels of SOC have been associated with improved physical and psychological health [42]. Studies have reported positive associations between SOC and the frequency of dental check-ups, positive oral health related behaviours and OHRQoL among non-Indigenous populations [43–46]. The same mechanism could be present and possibly amplified among Indigenous populations, who in general experience high levels of stress and social disadvantage, and reduced access to dental and other health services [47]. Thus, participants experiencing interpersonal racism may have a lower SOC that might consequently lead to poorer OHRQoL. Culturally-sensitive strategies that encompass Indigenous peoples' understanding of SOC are needed to plan such interventions, as it may be an important concept to target in dental health promotion strategies among Indigenous individuals.

We found that although the frequency of interpersonal racism was higher in males, the association between interpersonal racism and OHRQoL was strongest among females. Previous studies demonstrate that discriminaton is often compounded in Indigenous women, reflecting the combined effects of experiences of racism in daily activities, exclusion from economic opportunities and gender-based violence [29, 48]. Our findings add weight to this evidence demonstrating that interpersonal racism may significantly deteriorate OHRQoL among Indigenous women, more so than among Indigenous men. We speculate that this may be linked to a higher proportion of psychological distress among Indigenous women, which has been consistently reported in national health surveys of Aboriginal and Torres Strait Islander peoples [49]. Thus, experiences of racism and possible negative effects on psychological well-being may be compounded by the disproportionately higher levels of distress experienced by Indigenous women. Further research that explores the possible mediating or modifying effects of psychological distress and gender in the relationships between interpersonal racism and poor oral health is warranted. Further, our findings suggest a need for both a culturally- and gender-sensitive approach when implementing strategies to reduce oral health inequalities among Indigenous peoples.

The assessment of interpersonal racism in this study involved nine items, which gave us the flexibility to assess the frequency of interpersonal racism experienced in different situations (1–3 places or 4–9 places). In the crude analyses, we found that the poorest OHRQoL was observed among those who reported higher levels of interpersonal racism (e.g. in 4–9 settings). Increased odds of poor OHRQoL were also observed in those who reported lower levels of interpersonal racism, but to a lesser degree. However, in the adjusted analyses, the odds of poor OHRQoL were comparable  among those reporting experiencing low and high levels of racism, indicating that the negative effects of interpersonal racism on OHRQoL were felt irrespective of whether an individual experienced racism in one or many settings.  Notably, our effect sizes are similar to the strength of associations reported between interpersonal racism and general and oral health issues [5, 29, 50].

This is the first study to examine the association between interpersonal racism and OHRQoL among a large sample of Indigenous Australians. Established, validated instruments were used to capture both experiences of interpersonal racism and OHRQoL. Other strengths include use of Indigenous staff in all aspects of study management and a governance structure that meant the research was guided by an Indigenous Reference Group who provided cultural oversight. Our study findings should, however, be interpreted carefully as several limitations exist. First, participants were not representative of the entire Australian Aboriginal and Torres Strait Islander population, thus findings may not be generalizable. Second, the cross-sectional nature of data collection limits examination of temporality, and any causal assumptions. Third, a self-reported questionnaire was used to collect information on most of the variables therefore could be subject to recall and desirability bias. In addition, data on interpersonal racism was missing for 12% of the cohort. This may lead to a conservative bias, as it is possible that fears of disclosing experiences of interpersonal racism may have led some to avoid answering this question in the study. Thus, further studies, particularly with other Indigenous populations, are required to confirm these findings.

## Conclusion

In this cross-sectional study we identified a positive association between experiences of interpersonal racism and poor OHRQoL. The strength of the association was higher for females, but still present in males. The associations remained after adjustment for potential confounding due to socioeconomic position and use of dental services. Targeting the broader societal, cultural and historical determinants that influence interpersonal racism in Australia warrants an urgent implementation of microsocial policies that may buffer the experiences of racism among Indigenous Australians. These microsocial policies should target empowerment of Indigenous communities in oral health promotion decisions and improving SOC, and must be accompanied by the implementation of societal level policies (that are culturally sound and safe) to reduce interpersonal racism. This may, in turn, result in improved OHRQoL to support Indigenous Australians to be able to live lives free of dental pain and enjoying the full benefits of optimal oral health.

## Supplementary Information

**Additional file 1. Table S1**: Study questionnaire. **Table S2**: 14 items OHIP scale. **Fig. S3**: Goodness of fit.

## Data Availability

The original data, including all clinical and epidemiological data, used in this work will be made available upon reasonable request. Requests should be directed to the corresponding author.

## Abbreviations

OHRQoL: Oral health related quality of life
OHIP-14: Oral health impact profile
OPSCC: Oropharyngeal squamous cell carcinoma
MIRE: Measure of Indigenous racism experiences
OR: Odds ratio
AOR: Adjusted odds ratio
CI: Confidence interval
SOC: Sense of coherence

## Competing of interests

Dr. Anna Ali is supported by a Divisional Scholarship from The University of Adelaide. Associate Professor Alice Rumbold is a Principal Research Fellow at South Australian Health and Medical Research Institute. Dr. Kostas Kapellas is a research officer at the Australian Research Centre for Population Oral Health (ARCPOH), The University of Adelaide. Dr. Zohra Lassi is a NHMRC Early Career Research Fellow. Director Lisa Jamieson is a Professor at the Australian Research Centre for Population Oral Health (ARCPOH), The University of Adelaide.
