# Supplementary material for: The impact of interpersonal racism on oral health related quality of life among Indigenous South Australians: a cross-sectional study
Source: BMC Oral Health. 2021 Feb 4;21:46. doi: 10.1186/s12903-021-01399-1 (PMC7860008; doi:10.1186/s12903-021-01399-1)
Supplement: Supplementary file 1 — Additional file 1. Table S1: Study questionnaire. Table S2: 14 items OHIP scale. Fig. S3: Goodness of fit. [file 12903_2021_1399_MOESM1_ESM.docx]

**SUPPLEMENTARY DOCUMENT**

The impact of racism on oral health related quality of life among Indigenous South Australians; a cross-sectional study

**Supplemental tables and figures**

S1: Study questionnaire

S2: 14 items OHIP scale

S3: Goodness of fit

**Supplementary table: S1: Study questionnaire**

AUSTRALIAN RESEARCH CENTRE FOR POPULATION ORAL HEALTH

SCHOOL OF DENTISTRY

HPV and OROPHARYNGEAL CANCER (THROAT CANCER) STUDY

BASELINE QUESTIONNAIRE-(Section D-P)

1. Participant ID Number
2. Interviewer name
3. Date of interview (dd/mm/yy) _
4. Date of birth (dd/mm/yy)
5. Sex: Male/Female/Other

Please answer ALL of the sections in the survey, even if they do not seem to be directly relevant to *you*. Everything you tell us will be treated in strict confidence but you are free to leave any specific questions that you do not wish to answer.

If you are not sure of the correct answer, please give us your best estimate. We are asking many different people the same sets of questions and we are very interested in the different types of responses.

| **D. THESE QUESTIONS ARE ABOUT YOUR BACKGROUND. THERE ARE NO RIGHT OR WRONG ANSWERS** | | | | | |
| --- | --- | --- | --- | --- | --- |
| D1. Do you identify as being? | Aboriginal  1 | Torres Strait Islander  2 | Both  3 | Other  4 |  |

| D2. Level of education | No schooling | Primary school | High school | Trade or TAFE | University |
| --- | --- | --- | --- | --- | --- |
|  | 1 | 2 | 3 |   4 | 5 |

| D3. Income | Job  1 | Centrelink payment  2 | Other  3 |
| --- | --- | --- | --- |

| D4. Health Care Card | Yes  1 | No  2 | Don’t know  3 |
| --- | --- | --- | --- |

| D5. How many **people** stayed in the house last night? |  |  |  | ***Write the***  ***number in this box*** |
| --- | --- | --- | --- | --- |

| D6. Do you own a car? | Yes  1 | | No  2 | | Don’t know  3 | |
| --- | --- | --- | --- | --- | --- | --- |
| D7. Do you borrow a car? | Yes  1 | | No  2 | | Don’t know  3 | |
| **E. THESE QUESTIONS ARE ABOUT YOUR ALCOHOL AND TOBACCO CONSUMPTION** | | | | | | |
| *If you have never smoked or have smoked fewer than 100 cigarettes in your lifetime go to question E7.* | | | | | | |
| E1. Have you smoked more than 100 cigarettes in your lifetime? | | Yes  1 | | No  2  (If no please go to question E7) | | Rather not say  3 |
| *If you* ***currently*** *smoke cigarettes please answer Q E2-Q E3* | | | | | | |
| E2. For how long have you smoked cigarettes? | | Years Months | | | | |
| E3. On a usual day how many cigarettes do you smoke? | | Write the number in this box | | | | |
| *If you are a* ***former*** *smoker please answer Q E4 –Q E6* | | | | | | |
| E4. How long ago did you stop smoking cigarettes? | | Years Months | | | | |

| E5. For how long had you smoked cigarettes? | | Years Months | | | | | | | | | |
| --- | --- | --- | --- | --- | --- | --- | --- | --- | --- | --- | --- |
| E6. On a usual day, how many cigarettes did you smoke? | | | Write the number in this box | | | | | | | | |
| E7. Do you chew tobacco/ pituri? | | Yes  1 | | | | No  2 | | | Rather not say  3 | | |
| *If you have never had alcohol in your lifetime please go to question E10* | | | | | | | | | | | |
| E8. How often do you drink alcohol? | Daily  1 | | | Weekly  2 | | | Monthly  3 | | | Never  4 | |
| E9. How much alcohol do you drink per week? | 20+ Alcoholic drinks  1 | | | 8–19 Alcoholic drinks  2 | | | 1–7 Alcoholic drinks  3 | | | No-alcoholic drinks  4 | |
| E10. Which of the following best describes your non- tobacco substance smoking status  eg Vape/e-cigarette? | I currently smoke non-tobacco substances  1 | | | | I don’t smoke non-tobacco substances now but I used to  2 | | | | I have never smoked non- tobacco substances  3 | | |
| E11. Which of the following best describes your  recreational drug use? eg marijuana | I currently use recreational drugs  1 | | | | I don’t use recreational drugs now but I used to  2 | | | | I have never used recreational drugs  3 | | |
| **F. THESE QUESTIONS ARE ABOUT YOUR HUMAN PAPILLOMA VIRUS (HPV) STATUS. HPV IS A VERY COMMON SEXUALLY TRANSMITTED INFECTION THAT MOST AUSTRALIANS EXPERIENCE AT SOME TIME IN THEIR LIVES.** | | | | | | | | | | | |
| F1. Have you ever been diagnosed with having HPV? | | | | | | | | Yes  1 | No  2 | | Don’t know  3 |
| F2. Have you ever received a vaccination for HPV? | | | | | | | | Yes  1 | No  2 | | Don’t know  3 |

| F2a. If yes to F2, how many injections for the HPV vaccination did you receive? (the full amount is 3). | | Write the number in this box | | |
| --- | --- | --- | --- | --- |
| F3. Have you ever had your tonsils taken out? | Yes  1 | | No  2 | Don’t know  3 |

| F3a. If yes to F3, what was the age that you had your tonsils taken out? |  |  | Write the number in this box |
| --- | --- | --- | --- |

| **G. SEXUAL HEALTH BEHAVIOURS; The next section includes private questions about your personal life. These questions are important to understand possible links between life style and oral infections, including HPV.** | | | | | | | | | |
| --- | --- | --- | --- | --- | --- | --- | --- | --- | --- |
| G1. Altogether, in your life so far, how many people have you kissed passionately on the mouth?  (‘Passionate kissing’ refers to open- mouthed kissing) | None  1 | 1  2 | 2 to 3  3 | 4 to 7  4 | 8 to 15  5 | | 16 to 28  6 | 29 or more  7 |  |
| G2. Have you ever given oral sex?  (‘Oral sex’ refers to a man’s or woman’s mouth on a partner’s genital area) | No (go to G6)  1 | | | | | Yes (go to next question)  2 | | |  |

| G3.a How old were you when you first gave oral sex? | <16yrs (go to G4)  1 | =/ >16yrs (go to G3b)  2 |
| --- | --- | --- |

| G3.b How old? (if =/> 16yrs) |  |  | Years |
| --- | --- | --- | --- |

| G4. Altogether, in your life so far, how many people have you given oral sex to? | None  1 | 1  2 | | 2 to 3  3 | | 4 to 7  4 | 8 to 15  5 | | 16 to 28  6 | 29 or more  7 | | |
| --- | --- | --- | --- | --- | --- | --- | --- | --- | --- | --- | --- | --- |
| G5. When you give oral sex, how often is a condom or dental dam used?  (‘Dental dam’ refers to a thin square piece of latex used for protection during oral-vaginal or oral-anal sex) | Never  1 | | Seldom  2 | | | Occasionally  3 | | Frequently  4 | | | Very frequently  5 | Always  6 |
| G6. Have you ever received oral sex? | No (go to G10)  1 | | | | Yes (go to next question)  2 | | | | | | | |

| G7.a How old were you when you first received oral sex? | <16yrs (go to G8)  1 | =/ >16yrs (go to G7b)  2 |
| --- | --- | --- |

| G7.b How old? (if =/ > 16yrs) |  |  | Years |
| --- | --- | --- | --- |

| G8. Altogether, in your life so far, how many people have you received oral sex from? | None  1 | 1  2 | 2 to 3  3 | 4 to 7  4 | 8 to 15  5 | 16 to 28  6 | 29 or more  7 |
| --- | --- | --- | --- | --- | --- | --- | --- |

| G9. When you receive oral sex, how often is a condom or dental dam used?  (‘Dental dam’ refers to a thin square piece of latex used for protection during oral-vaginal or oral-anal sex) | Never  1 | Seldom  2 | | Occasionally  3 | Frequently  4 | Very frequently  5 | Always  6 |
| --- | --- | --- | --- | --- | --- | --- | --- |
| G10. Have you ever had sexual intercourse with another person?  (‘Sexual intercourse’ refers to vaginal intercourse and anal intercourse only. ‘Sexual intercourse’ does not refer to oral sex) | No (go to G13)  1 | | Yes (go to next question)  2 | | | | |

| G11.a How old were you when you first had sexual intercourse? | <16yrs (go to G12)  1 | =/ >16yrs (go to G11b)  2 |
| --- | --- | --- |

| G11.b How old? (if =/ > 16yrs) |  |  | **Years** |
| --- | --- | --- | --- |

| G12. Altogether, in your life so far, how many people have you had sexual intercourse with? | None  1 | | 1  2 | 2 to 3  3 | | | 4 to 7  4 | | 8 to 15  5 | | 16 to 28  6 | | 29 or more  7 |
| --- | --- | --- | --- | --- | --- | --- | --- | --- | --- | --- | --- | --- | --- |
| G13. In your lifetime, who have you had sexual contact with? | Men only  1 | Mostly men, sometimes women  2 | | | Equally men and women  3 | | | Women only  4 | | Mostly women, sometimes men  5 | | | |
| G14. How would you describe your current relationship status? | Currently in stable, long-term relationship  1 | | | | | Currently in short-term relationships  2 | | | | | | Currently single  3 | |

| **H. SELF-RATED HEALTH** | | | | | |
| --- | --- | --- | --- | --- | --- |
| H1. Would you rate your general health as: | Excellent  1 | Very good  2 | Good  3 | Fair  4 | Poor  5 |
| H2. Would you rate your oral health as: | Excellent  1 | Very good  2 | Good  3 | Fair  4 | Poor  5 |

| **I. DENTAL BEHAVIOURS** | | |
| --- | --- | --- |
| I1. When did you last see a dentist: | Less than one year ago  1 | More than one year ago  2 |
| I2. What is your usual reason for seeing a dentist: | Problem  1 | Check-up  2 |

| I3. During the last year, have you not gone to the dentist because of cost: | Yes  1 | | | No  2 | | |
| --- | --- | --- | --- | --- | --- | --- |
| I4. How hard would it be for you to pay a $100 dental bill: | Not hard at all  1 | Not very hard  2 | A little bit hard  1 | | Very hard  2 | Could not pay  2 |

| **J. THE QUESTIONS BELOW ASK ABOUT TROUBLES THAT PEOPLE MAY HAVE IN DAILY LIFE BECAUSE OF DENTAL PROBLEMS.** | | | | | |
| --- | --- | --- | --- | --- | --- |
| *How often during the last year….* | **Please tick ONE box that best describes your experience** | | | | |
| J1. … have you had trouble **pronouncing (or saying)** any **words** because of problems with your teeth, mouth or false teeth? | Very often  1 | Fairly often  2 | Occasionally  3 | Hardly ever  4 | Never  5 |
| J2. … have you felt that your **sense of taste** has worsened because of problems with your teeth, mouth or false teeth? | Very often  1 | Fairly often  2 | Occasionally  3 | Hardly ever  4 | Never  5 |
| J3. … have you had **painful aching** in your mouth? | Very often  1 | Fairly often  2 | Occasionally  3 | Hardly ever  4 | Never  5 |
| J4. … have you found it **uncomfortable to eat any foods** because of problems with your teeth, mouth or false teeth? | Very often  1 | Fairly often  2 | Occasionally  3 | Hardly ever  4 | Never  5 |
| J5. … have you been **self- conscious** because of problems with your teeth, mouth or false teeth? | Very often  1 | Fairly often  2 | Occasionally  3 | Hardly ever  4 | Never  5 |
| J6. … have you **felt tense** because of problems with your teeth, mouth or false teeth? | Very often  1 | Fairly often  2 | Occasionally  3 | Hardly ever  4 | Never  5 |
| J7. … has your **diet been unsatisfactory** because of problems with your teeth, mouth or false teeth? | Very often  1 | Fairly often  2 | Occasionally  3 | Hardly ever  4 | Never  5 |
| J8. … have you had to **interrupt meals** because of problems with your teeth, mouth or false teeth? | Very often  1 | Fairly often  2 | Occasionally  3 | Hardly ever  4 | Never  5 |

| J9. … have you found it **difficult to relax** because of problems with your teeth, mouth or false teeth? | Very often  1 | Fairly often  2 | Occasionally  3 | Hardly ever  4 | Never  5 |
| --- | --- | --- | --- | --- | --- |
| J10. … have you been a bit **embarrassed** because of problems with your teeth, mouth or false teeth? | Very often  1 | Fairly often  2 | Occasionally  3 | Hardly ever  4 | Never  5 |
| J11. … have you been **irritable with other people** because of problems with your teeth, mouth or false teeth? | Very often  1 | Fairly often  2 | Occasionally  3 | Hardly ever  4 | Never  5 |
| J12. … have you had **difficulty doing your usual jobs** because of problems with your teeth, mouth or  false teeth? | Very often  1 | Fairly often  2 | Occasionally  3 | Hardly ever  4 | Never  5 |

| J13. … have you felt that life in general was **less satisfying** because of problems with your teeth, mouth or  false teeth? | Very often  1 | Fairly often  2 | Occasionally  3 | Hardly ever  4 | Never  5 |
| --- | --- | --- | --- | --- | --- |
| J14. … have you been totally **unable to function** because of problems with your teeth, mouth or false teeth? | Very often  1 | Fairly often  2 | Occasionally  3 | Hardly ever  4 | Never  5 |

| **K. THIS SECTION ASKS ABOUT YOUR GENERAL QUALITY OF LIFE; WHICH OF THE FOLLOWING STATEMENTS BEST DESCRIBES YOUR HEALTH TODAY REGARDING** | | | | | |
| --- | --- | --- | --- | --- | --- |
| K1. Mobility | I have no problems in walking about | I have slight problems in walking about | I have moderate problems in  walking about | I have severe problems in walking about | I am unable to walk about  5 |
|  | 1 | 2 | 3 | 4 |  |
|  | I have no | I have slight | I have | I have severe | I am unable to |
|  | problems | problems | moderate | problems | wash or dress |
|  | washing or | washing or | problems | washing or | myself |
| K2. Self-care | dressing myself | dressing myself | washing or dressing myself | dressing myself | 5 |
|  | 1 | 2 | 3 | 4 |  |
| K3. Usual activities, for example, work, study housework, family or leisure activities | I have no problems doing my usual activities  1 | I have slight problems doing my usual activities  2 | I have moderate problems doing my usual activities  3 | I have severe problems doing my usual activities  4 | I am unable to do my usual activities  5 |

| K4. Pain/Discomfort | I have no pain or discomfort  1 | I have slight pain or discomfort  2 | I have moderate pain or discomfort  3 | I have severe pain or discomfort  4 | I have extreme pain or discomfort  5 |
| --- | --- | --- | --- | --- | --- |

| K5. Anxiety/Depression | I am not anxious or depressed  1 | I am slightly anxious or depressed  2 | I am moderately anxious or depressed  3 | I am severely anxious or depressed  4 | I am extremely anxious or depressed  5 |
| --- | --- | --- | --- | --- | --- |

**L. THIS SECTION ASKS ABOUT YOUR FEELINGS WHEN OTHERS IN YOUR FAMILY OR COMMUNITY GET CANCER/THE BIG C.**

**WHEN OTHERS IN YOUR FAMILY/COMMUNITY GET CANCER/ THE BIG C, DO YOU FEEL? …………..**

| L1. | Anger | Yes  1 | No  2 |
| --- | --- | --- | --- |
| L2. | Frustration | Yes  1 | No  2 |
| L3. | Sadness… | Yes  1 | No  2 |

| L4. Guilt | Yes  1 | No  2 |
| --- | --- | --- |

| L5. Revenge | Yes  1 | No  2 |
| --- | --- | --- |

| L4. Fear | Yes  1 | No  2 |  |
| --- | --- | --- | --- |
| L5. Other; please explain |  | | |

**M. THIS SECTION ASKS ABOUT YOUR CULTURAL IDENTIFY AND EXPERIENCES OF RACISM OR DISCRIMINATION.**

| M1. Do you know a lot about your Aboriginal/Torres Strait Islander culture? | Lots  1 | Fair bit  2 | Little bit  3 | Not much  4 |
| --- | --- | --- | --- | --- |
| M2. Do you identify with a tribal group, a language group or clan? | Yes (Specify )  1 | | No  2 | Don’t know  3 |
| M3. To you, is being Aboriginal/Torres Strait Islander… | The most important thing (central to who you are)  1 | Important, but not the only thing  2 | Something you don’t know enough about and want to know more about  3 | Something you don’t think about  4 |
| M4. Do you feel like you know a lot about white fella ways? | Lots  1 | Fair bit  2 | Little bit  3 | Not much  4 |
| M5. Do you have a strong family who help each other? | Always  1 | Most times  2 | Sometimes  3 | Not really  4 |

**N. IN THE LAST 12 MONTHS, HAVE YOU EVER FELT THAT YOU HAVE BEEN TREATED UNFAIRLY IN ANY OF THE FOLLOWING WAYS BECAUSE OF YOUR IDENTITY AS AN ABORIGINAL/TORRES STRAIT ISLANDER PERSON.**

|  | **Strongly Disagree** |  |  | **Strongly Agree** | |
| --- | --- | --- | --- | --- | --- |
| N.1 Applying for work or when at work. | □1 | □2 | □3 | □4 | □5 |
| N.2 At home, by neighbours, or at somebody else’s house. | □1 | □2 | □3 | □4 | □5 |
| N.3 At school, university, training course, or other educational setting. | □1 | □2 | □3 | □4 | □5 |
| N.4 While doing any sporting, recreational or leisure activities. | □1 | □2 | □3 | □4 | □5 |
| N.5 By the police, security people, lawyers or in a court of law. | □1 | □2 | □3 | □4 | □5 |
| N.6 By doctors, dentists, nurses or other staff at hospitals, dental clinics or  doctor surgeries. | □1 | □2 | □3 | □4 | □5 |
| N.7 By staff of government agencies. | □1 | □2 | □3 | □4 | □5 |
| N.8 When seeking any other services. | □1 | □2 | □3 | □4 | □5 |
| N.9 By members of the general public. | □1 | □2 | □3 | □4 | □5 |
| N.10 Any other situation. (*please specify*) | □1 | □2 | □3 | □4 | □5 |

**O. PAIN. THIS SECTION ASKS ABOUT YOUR EXPERIENCES OF PAIN BOTH NOW AND IN THE COURSE OF YOUR LIFE.**

| **O1.** Do you now have significant pain that has lasted 6 months or more? ***If NO, skip to O2*** | Yes  1 | No  2 |
| --- | --- | --- |

| O1a. How severe would you rate that pain, on a scale from 0-100, with 0 being “no pain” and 100 being “the maximum pain possible”? | Write the number in this box |
| --- | --- |
| O1b. How long has this pain lasted? | Write the number in this box |
| O1c. How many days a week do you experience this pain? | Write the number in this box |
| O1d. What is the source of this pain? | Write the answer in this box |

| **O2.** Do you have any other significant pain now that has lasted 6 months or more? ***If NO, skip to O3*** | Yes  1 | No  2 |
| --- | --- | --- |

| O2a. How severe would you rate that pain, on a scale from 0-100 with 0 being “no pain” and 100 being “the maximum pain possible”? | Write the number in this box |
| --- | --- |
| O2b. How long has this pain lasted? | Write the number in this box |
| O2c How many days a week do you experience this pain? | Write the number in this box |
| O2d. What is the source of this pain? | Write the answer in this box |

| **O3.** Do you now have significant pain that has lasted less than 6 months? ***If NO, skip to O4*** | Yes  1 | No  2 |
| --- | --- | --- |
| O3a. How severe would you rate that pain, on a scale from 0-100 with 0 being “no pain” and 100 being “the maximum pain possible”? | Write the number in this box | |
| O3b. How long has this pain lasted? | Write the number in this box | |
| O3c. How many days a week do you experience this pain? | Write the number in this box | |
| O3d. what is the source of this pain? | Write the answer in this box | |

| **O4.** Besides any pain just discussed, have you ever had significant pain? ***If NO, skip to section P*** | Yes  1 | No  2 |
| --- | --- | --- |
| O4a. How severe would you rate that pain, on a scale from 0-100 with 0 being “no pain” and 100 being “the maximum pain possible”? | Write the number in this box | |
| O4b. How long has this pain lasted? | Write the number in this box | |
| O4c. How many days a week do you experience this pain? | Write the number in this box | |
| O4d. What is the source of this pain? | Write the answer in this box | |

**P. FEAR OF PAIN: THIS SECTION HAS QUESTIONS ASKING ABOUT ANY FEAR OF PAIN THAT YOU MIGHT EXPERIENCE. THE ITEMS LISTED BELOW DESCRIBE PAINFUL EXPERIENCES. PLEASE LOOK AT EACH ITEM AND THINK ABOUT HOW FEARFUL YOU ARE OF EXPERIENCING THE PAIN ASSOCIATED WITH EACH ITEM. IF YOU HAVE NEVER EXPERIENCED THE PAIN OF A PARTICULAR ITEM, PLEASE ANSWER ON THE BASIS OF HOW FEARFUL YOU EXPECT YOU WOULD BE IF YOU HAD SUCH AN EXPERIENCE. CIRCLE ONE NUMBER FOR EACH ITEM BELOW TO RATE YOUR FEAR OF PAIN IN RELATION TO EACH EVENT.**

**I FEAR THE PAIN** associated with:

| P1. Breaking your arm | Not at all  1 | A little  2 | A fair amount  3 | Very much  4 | Extreme  5 |
| --- | --- | --- | --- | --- | --- |
| P2. Having a foot doctor remove a wart from your foot with a sharp instrument. | Not at all  1 | A little  2 | A fair amount  3 | Very much  4 | Extreme  5 |
| P3. Getting a paper-cut on your finger. | Not at all  1 | A little  2 | A fair amount  3 | Very much  4 | Extreme  5 |
| P4. Receiving an injection in your mouth. | Not at all  1 | A little  2 | A fair amount  3 | Very much  4 | Extreme  5 |
| P5. Getting strong soap in both your eyes while bathing and showering. | Not at all  1 | A little  2 | A fair amount  3 | Very much  4 | Extreme  5 |
| P6. Having someone slam a heavy car door on your hand. | Not at all  1 | A little  2 | A fair amount  3 | Very much  4 | Extreme  5 |
| P7. Gulping a hot drink before it has cooled. | Not at all  1 | A little  2 | A fair amount  3 | Very much  4 | Extreme  5 |
| P8. Receiving an injection in your hip/buttocks. | Not at all  1 | A little  2 | A fair amount  3 | Very much  4 | Extreme  5 |
| P9. Falling down a flight of concrete stairs. | Not at all  1 | A little  2 | A fair amount  3 | Very much  4 | Extreme  5 |

**THANK YOU FOR TAKING THE TIME TO ANSWER OUR QUESTIONS**

**PLEASE SHARE ANYTHING ELSE YOU WOULD LIKE TO TELL US IN THE SPACE BELOW.**

**Supplementary table S2:** 14 items OHIP scale

| Have you had trouble pronouncing (or saying) any words because of problems with your teeth, mouth or false teeth? |
| --- |
| Have you felt that your sense of taste has worsened because of problems with your teeth, mouth or false teeth? |
| Have you had painful aching in your mouth? |
| Have you found it uncomfortable to eat any foods because of problems with your teeth, mouth or false teeth? |
| Have you been self- conscious because of problems with your teeth, mouth or false teeth? |
| Have you felt tense because of problems with your teeth, mouth or false teeth? |
| Has your diet been unsatisfactory because of problems with your teeth, mouth or false teeth? |
| Have you had to interrupt meals because of problems with your teeth, mouth or false teeth? |
| Have you found it difficult to relax because of problems with your teeth, mouth or false teeth? |
| Have you been a bit embarrassed because of problems with your teeth, mouth or false teeth? |
| Have you been irritable with other people because of problems with your teeth, mouth or false teeth? |
| Have you had difficulty doing your usual jobs because of problems with your teeth, mouth or false teeth? |
| Have you felt that life in general was less satisfying because of problems with your teeth, mouth or false teeth? |
| Have you been totally unable to function because of problems with your teeth, mouth or false teeth? |

Responses: very often (4), often (3), occasionally (2), hardly even (1) and never (0)

**Supplementary figures: S3: Goodness of fit**

**Model 1: Goodness of fit: Model 2: Goodness of fit:**

**
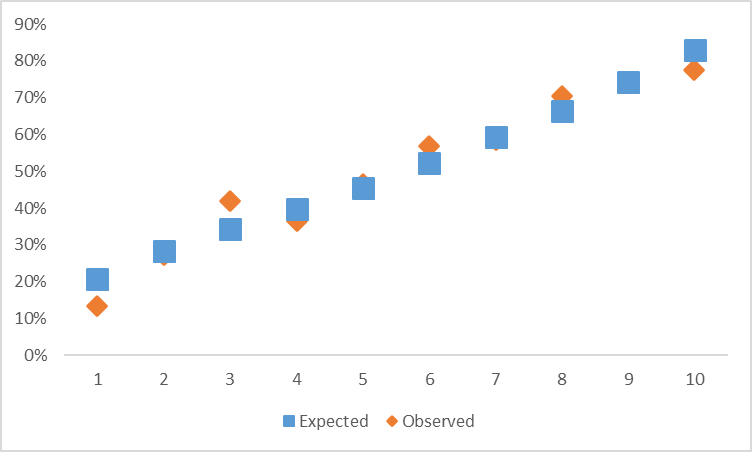
**

**
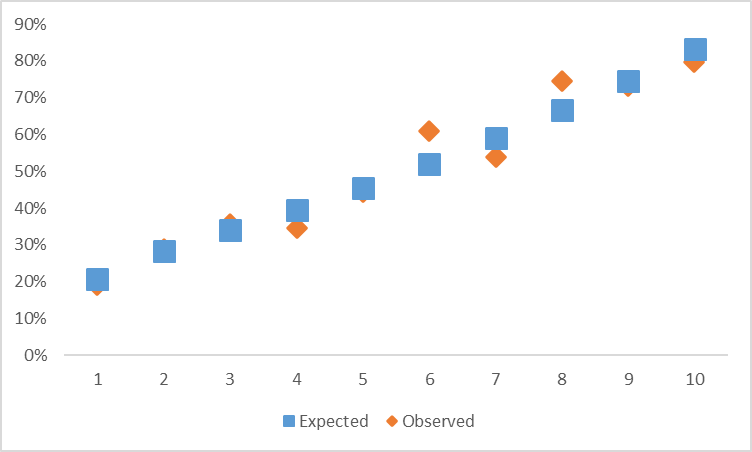
Model 3: Goodness of fit: Model 4: Goodness of fit**

**
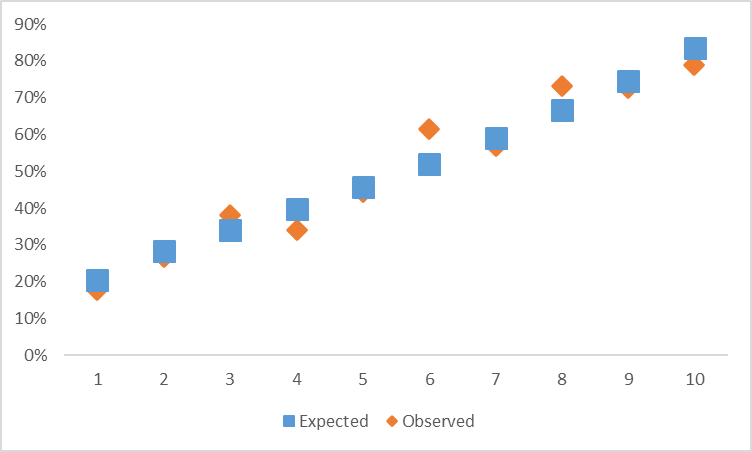
**
